# Aroma Profile of Montepulciano d'Abruzzo Wine Fermented by Single and Co-culture Starters of Autochthonous *Saccharomyces* and Non-*saccharomyces* Yeasts

**DOI:** 10.3389/fmicb.2016.00610

**Published:** 2016-04-28

**Authors:** Rosanna Tofalo, Francesca Patrignani, Rosalba Lanciotti, Giorgia Perpetuini, Maria Schirone, Paola Di Gianvito, Daniel Pizzoni, Giuseppe Arfelli, Giovanna Suzzi

**Affiliations:** ^1^Faculty of BioScience and Technology for Food, Agriculture and Environment, University of TeramoMosciano Sant'Angelo, Italy; ^2^Department of Agricultural and Food Sciences, University of BolognaBologna, Italy

**Keywords:** aroma compounds, autochthonous yeast strains, *Saccharomyces cerevisiae*, non-*Saccharomyces*, Montepulciano d'Abruzzo wine

## Abstract

Montepulciano d'Abruzzo is a native grape variety of *Vitis vinifera* L., grown in central Italy and used for production of high quality red wines. Limited studies have been carried out to improve its enological characteristics through the use of indigenous strains of *Saccharomyces cerevisiae*. The main objective of the present work was to test two indigenous strains of *S. cerevisiae* (SRS1, RT73), a strain of *Starmerella bacillaris* (STS12), one of *Hanseniaspora uvarum* (STS45) and a co-culture of *S. cerevisiae* (SRS1) and *S. bacillaris* (STS12), in an experimental cellar to evaluate their role in the sensory characteristic of Montepulciano d'Abruzzo wine. A *S. cerevisiae* commercial strain was used. Fermentations were conducted under routine Montepulciano d'Abruzzo wine production, in which the main variables were the yeast strains used for fermentation. Basic winemaking parameters, some key chemical analysis and aroma compounds were considered. *S. cerevisiae* strain dynamics during fermentation were determined by molecular methods. The musts inoculated with the co-culture were characterized by a faster fermentation start and a higher content of glycerol after 3 days of fermentation, as well as the musts added with strains *S. bacillaris* (STS12) and *H. uvarum* (STS45). At the end of fermentation the parameters studied were quite similar in all the wines. Total biogenic amines (BA) content of all the wines was low. Ethanolamine was the predominant BA, with a concentration ranging from 21 to 24 mg/l. Wines were characterized by esters and alcohols. In particular, 2-phenylethanol, 3-methylbut-1-yl methanoate, and ethyl ethanoate were the major aroma volatile compounds in all wines. Statistical analysis highlighted the different role played by aroma compounds in the differentiation of wines, even if it was impossible to select a single class of compounds as the most important for a specific yeast. The present study represents a further step toward the use of tailored autochthonous strains to impart the specific characteristics of a given wine which are an expression of a specific terroir.

## Introduction

Wine fermentations constitute complex microbial ecosystems consisting of highly dynamic yeast communities which play a key role in shaping wine quality (Fleet, 2003). This complex array of relations influences the nutritional, hygienic, and aromatic features of the product through the consecutive growth and death of different species and strains within each species, during the fermentation process (Fleet, 2003; Liu et al., 2015). Many studies have been focused on the nature of these relations improving the knowledge about ecology, physiology, biochemistry, and molecular biology of the microrganisms involved in wine fermentation process underlying the ecological complexity and variability of these fermentations that extend beyond the species level (for a review see Liu et al., 2015).

Yeasts mainly impact on the wine flavor producing a large array of volatile substances (Howell et al., 2006). In this context the existing commercial yeast strains present some limits, especially because they reduce the uniqueness of wine bouquet (Alves et al., 2015). In fact, different yeast species and even different genotypes of *Saccharomyces cerevisiae* produce different wine aroma profiles (Alves et al., 2015; Barbosa et al., 2015; Vernocchi et al., 2015). This awareness opened new issues to meet wine-maker demand for “special yeasts for special traits” (Schuller and Casal, 2005; Sadoudi et al., 2012). Recently, the role of indigenous yeast strains has gained importance, as a tool to impart regional characters to wines. Indeed, the use of a “microarea-specific” starter culture highlighted the association between the volatile profile of wine and the geographical origin of the yeast used for the fermentation process (Tufariello et al., 2014).

The role of non-*Saccharomyces* (NS) yeasts in winemaking has been re-evaluated, leading to a more complex “flavor phenotype” producing more than 1300 volatile compounds e.g., esters, higher alcohols, acids, and monoterpenes (Swiegers et al., 2005; for a review see Jolly et al., 2014). Moreira et al. (2005) and Medina et al. (2013) demonstrated that *Hanseniaspora uvarum* increased the quantity of some desirable compounds, such as higher alcohols and esters, while Rantsiou et al. (2012) showed that inoculation with selected couples of *S. cerevisiae* and *Starmerella bacillaris* resulted in a decrease of about 0.3 g/l of acetic acid, maintaining high ethanol and glycerol levels.

Montepulciano d'Abruzzo is a red wine grape variety of *Vitis vinifera* L., grown widely in central Italy, most notably in Abruzzo, Marche, and Molise regions. However, it is mainly identified with Abruzzo, the region in which it is also the most common and cultivated red variety for over two centuries. The first report of the Montepulciano grape in Abruzzo is found in “*Saggio Itinerario Nazionale nel Paese dei Peligni*,” written by Torcia (1972). It currently accounts for around 50% of the regional vineyard, that is, about 18.500 hectares (Regione Abruzzo, http://www.regione.abruzzo.it/). Montepulciano d'Abruzzo is used for production of high quality red wines characterized by fruity notes (apple, pear, cherry, etc.). The most famous example is Montepulciano d'Abruzzo “Colline Teramane” DOCG wine (recognition in 2003) produced in the Teramo province.

Despite the economic importance of Montepulciano d'Abruzzo “Colline Teramane” few studies have been performed to identify its enological characteristics. In a previous study Tofalo et al. (2011) highlighted that the major NS yeasts present during must fermentation of Montepulciano cultivar were *H. uvarum, Metschnikowia fructicola*, and *S. bacillaris*, representing 43, 31, and 11%, respectively, of the total NS population isolated. Selected strains of *H. uvarum* (STS45), *S. bacillaris (*STS12), and *S. cerevisiae* (SRS1 and RT73) were then studied to evaluate their fermentation performance and interactions in microvinifications (Suzzi et al., 2012a,b).

The aim of this study was to establish the role and the inter-strains variability of two indigenous strains of *S. cerevisiae* (SRS1, RT73), a strain of *S. bacillaris* (STS12), one of *H. uvarum* (STS45) and a co-culture of *S. cerevisiae* (SRS1), and *S. bacillaris* (STS12) in shaping Montepulciano d'Abruzzo wine aroma profile in an experimental cellar. A *S. cerevisiae* commercial strain was used. Vinifications were conducted under routine Montepulciano d'Abruzzo wine production. Basic winemaking parameters (residual sugar, glycerol, organic acids, etc.), biogenic amines (BA) and volatile metabolites were determined. *S. cerevisiae* strain dynamics were also determined by microsatellite analysis.

## Materials and methods

### Yeast strains and media

Non-*Saccharomyces* (*H. uvarum*, STS45 and *S. bacillaris*, STS12) and *S. cerevisiae* autochthonous strains (RT73 and SRS1) have been previously characterized for their oenological performances in Montepulciano d'Abruzzo microvinification trials (Suzzi et al., 2012a,b). A commercial strain (CS) of *S*. *cerevisiae* (Flower Fresh, Tecnofood, Pavia, Italy) was also used. All strains belong to the Culture Collection of the Faculty of BioScience and Technology for Food, Agriculture, and Environment (University of Teramo, Italy). Non-*Saccharomyces* and *S*. *cerevisiae* strains were routinely grown in YPD medium (1% w/v yeast extract, 2% w/v peptone, and 2% w/v glucose) for 48 h under aerobic conditions. All strains were stored at −80°C in YPD broth supplemented with glycerol (20% v/v final concentration; Sigma-Aldrich, Milan, Italy).

### Cellar vinifications

Vinifications were carried out in a cellar of Consorzio per la Ricerca Viticola ed Enologica in Abruzzo (CRIVEA), during the vintage 2011. Montepulciano d'Abruzzo must (235 g/l fermentable sugars, 8.17 titratable acidity (TTA) and pH 3.44) was separated in tanks of 50 l, after destemming and crushing and added with 100 mg/l potassium metabisulfite.The fermentations were performed in maceration with the skins. The tanks were inoculated with 10^6^ cells/ml from 24 h pre-cultures grown in the same pasteurized must. Two *S. cerevisiae* strains (SRS1, RT73), a strain of *S. bacillaris* (STS12), one of *H. uvarum* (STS45), and a co-culture of SRS1+STS12 were used to conduct fermentations. All fermentations were carried out in triplicate at room temperature (maximum temperature variation from 9 to 19°C). When the fermentation ended, the yeast lees were allowed to settle for 7 days and then wines were racked in 40 l tanks and stored at controlled temperature in the cellar for 3 months. Then the wines were placed into glass bottles (750 ml), crown-sealed, and stored at 15–20°C for up to 6 months until sensorial analyses were performed.

### Enumeration and yeast isolation

Total viable yeast counts were performed after 3, 5, 7, 10, and 15 days, using Wallerstein Laboratory Nutrient Agar (WLN, Oxoid, Milan, Italy), according to Pallmann et al. (2001).

### Analytical determinations

The main wine analytical components (ethanol, reducing sugar, pH, volatile acidity, TTA, citric, lactic, malic, and tartaric acids, glycerol) were determined using a FOSS WineScan (FT-120) rapid scanning Fourier Transform Infrared Spectroscopy with FOSS WineScan software version 2.2.1. Samples were firstly centrifuged at 8000 g for 10 min and then analyzed following the manufacturer's instructions.

### Microsatellite PCR fingerprinting

Total DNA was extracted directly from musts and wines using the PowerSoil DNA Isolation Kit (MoBio Laboratories). Ten milliliter of each sample were centrifuged to collect cells. The DNA was then extracted according to manufacturer's protocol. Quantification of total DNA was achieved using a VersaFluor fluorimeter and a Fluorescent DNA Quantitation Kit (Bio-Rad, Milan Italy). DNA was used as a template for microsatellite PCR fingerprinting, as described by Vaudano and Garcia-Moruno (2008). PCR amplifications were performed in a thermocycler (MyCycler, Bio-Rad Laboatories, Milan, Italy) with the following PCR programme: 4 min of initial denaturation at 94°C, 28 cycles of 30 s at 94°C, 45 s at 56°C, 30 s at 72°C and, finally, 10 min at 72°C. The products were run on a 2.5% (w/v) agarose gel 1 × TAE buffer at 100 V for 80 min. Gels were stained with ethidium bromide. 1-kb plus DNA ladder (Life Technologies, Milan, Italy) was used as marker for the gel normalization.

### Volatile profiles

Volatile compounds were determined by solid phase microextraction coupled with gas chromatography (GC/MS-SPME) according to Suzzi et al. (2012a). Molecule identification was based on comparison of their retention times with those of pure compounds (Sigma-Aldrich, Milan, Italy) analyzed in the same conditions. The identification was further confirmed by comparing mass spectra of compounds with those contained in the available database (NIST version 2005). The data were expressed as the relative peak area (%) calculated from head space SPME (HS/SPME) gas chromatograms of the identified peaks. All determinations were performed in triplicate.

### Biogenic amines determination

Biogenic amines (BA) were determined according to Manetta et al. (2016). BA were analyzed using an HPLC system consisting of an Alliance (Waters, Milford, MA, USA), equipped with a Waters 2695 separation module connected to a Waters 2996 photodiode array detector (PDA), set at 254 nm. A Supelcosil LC-18 column (5 μm particle size, 250 × 4.6 mm i.d.) from Sigma was used. The system was governed by Waters Empower personal computer software. All analyses were performed in triplicate.

### Sensory analysis

Sensory tests were performed at room temperature (20°C). Wine samples were coded with 3-digit numbers, were evaluated in triplicate and presented according to a completely randomized block design. Skilled judges (*n* = 13) were trained as stated in the ISO 8586-1: 1993 rules (ISO, 1993).

Descriptive analysis was carried out in only one session. Sensory profile was determined using nine descriptors (fruity, persistence, body, astringency, grassy, reduced, floral, tropical fruits, drupaceous fruits) as previously reported (Suzzi et al., 2012a). Samples were scored for selected descriptors on a 4 cm scale anchored with “low” and “high” intensity.

### Statistical analysis

All data were processed using Excel 2016 (Microsoft, USA) and MatLab 2009b (Mathworks, Natick, MA, USA) softwares. In particular, a Principal Component Analysis (PCA) was performed on SPME–GC data after auto-scaling. The volatile molecule data were used to build up a single matrix, which was submitted to a two-way hierarchical clustering analysis. A heat map, visualizing metabolite levels was then obtained in which values are represented by cell colored according to the *Z*-scores, where *Z* is the mean value of different vinifications with the same yeast strain (Ferrara et al., 2008; Serrazanetti et al., 2011). The significant differences of the main enological characteristics were determined by *F*-test.

## Results

### Viable counts and strain dynamics

In order to improve the quality of Montepulciano d'Abruzzo wine through the use of autochthonous wine yeasts, *Saccharomyces* and non-*Saccharomyces* strains isolated from the *terroir* “Colline Teramane” and characterized for their enological aptitudes (Suzzi et al., 2012a,b) were chosen for experimental cellar vinifications as reported in Materials and Methods. Six vinifications were carried out, two inoculated with single *S*. *cerevisiae* strains (SRS1 and RT73), two with single NS strains (*H*. *uvarum* STS45 and *S*. *bacillaris* STS12) and one with the simultaneous presence of SRS1 and STS12.

**Figure 1:**
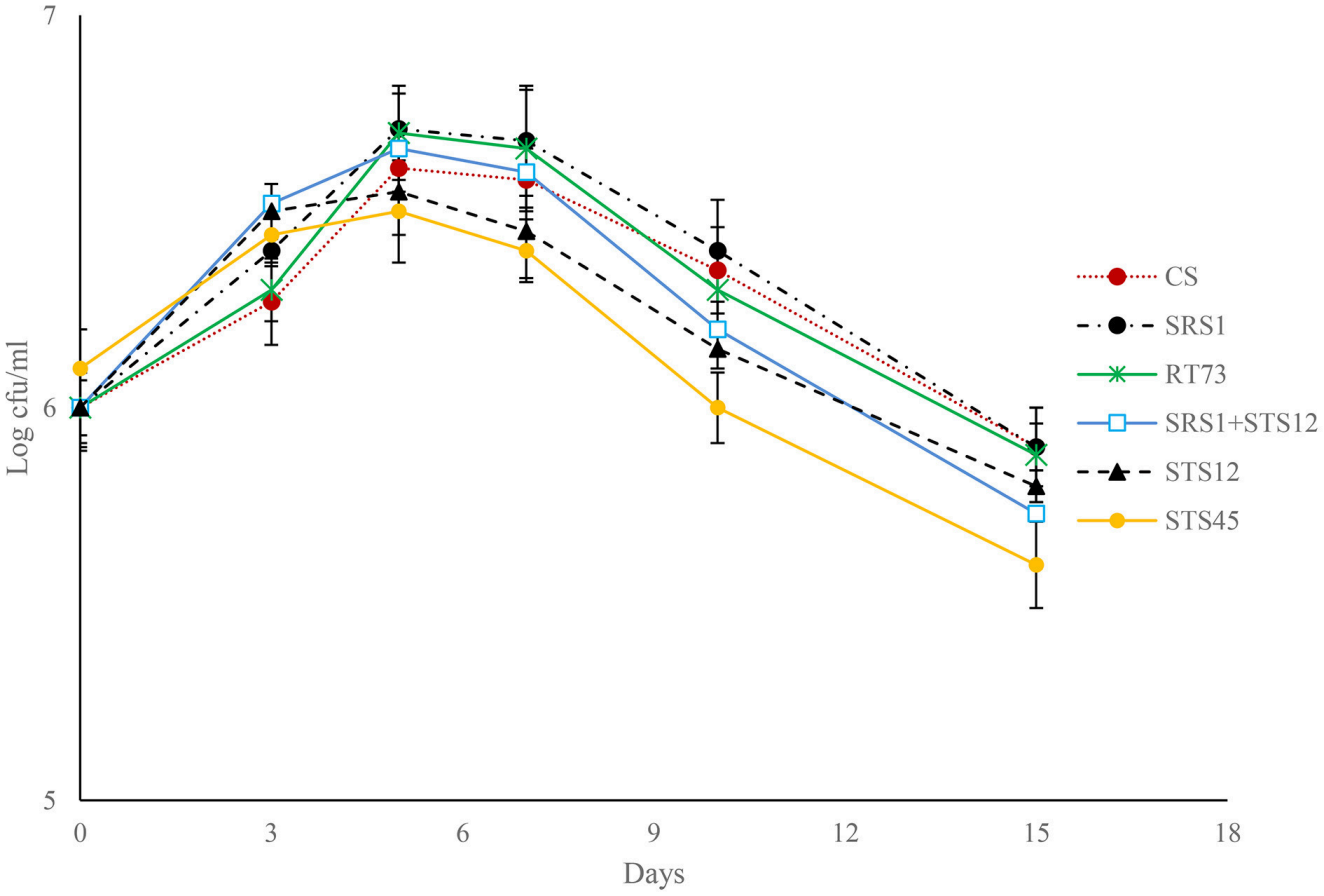
**Growth kinetic profiles of pure and mixed fermentation trials**.

Fermentation trials inoculated with SRS1, RT73, and the co-culture (SRS1+STS12) started the fermentation quickly (Figure 1), reaching higher values of viable cells after 5 days. At the end of fermentation lower values were observed in must inoculated with *S. bacillaris* STS12 and *H*. *uvarum* STS45, even if a faster growth was observed during the first fermentation days.

**Figure 2:**
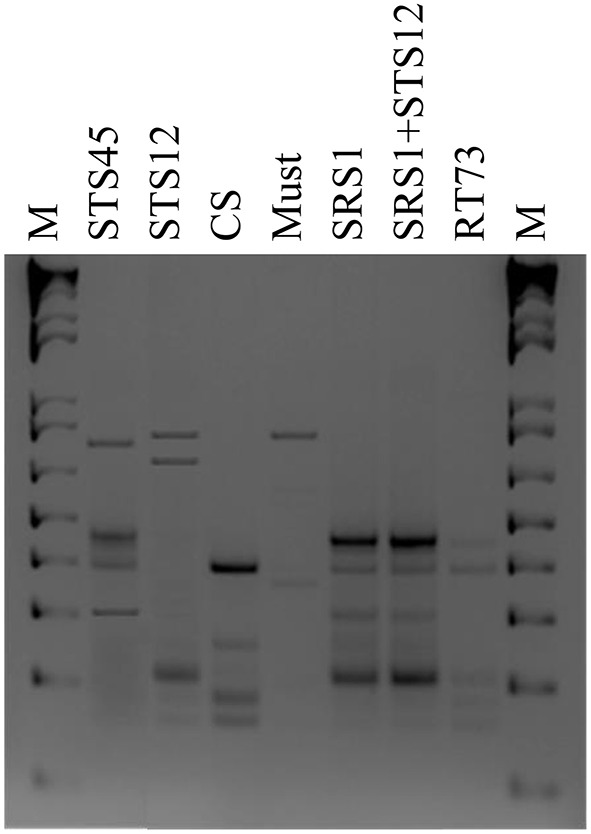
**Yeast strains electrophoretic patterns of microsatellite multiplex PCR (SC8132X, YOR267C and SCPTSY7) at the end of fermentation (15 days)**. Similar profiles were obtained after 3 days in inoculated fermentations. M: 1-kb plus DNA ladder (Life Technologies).

To verify the dominance of inoculated strains on natural yeast population in the must, microsatellite analysis on total DNAs was performed. The *S. cerevisiae* SRS1, RT73, and CS were present during the whole fermentation process, confirming a clear dominance of these *S*. *cerevisiae* strains (Figure 2). As expected more complex profiles were detected in Montepulciano d'Abruzzo must inoculated with *S. bacillaris* STS12 and *H. uvarum* STS45 probably due to the presence of different indigenous *S*. *cerevisiae* strains.

### Wine characteristics

During the first days of fermentation a higher production of ethanol by NS and mixed cultures was observed (Table 1), whereas no differences were registered at the end of fermentation. In fact in all the six different conditions, must fermentations were completed according to reducing sugar concentration. The NS strains formed higher levels of glycerol up to 3.39 g/l after 3 days of fermentation, whereas the *S. cerevisiae* strains ranged from 1.38 to 2.20 g/l. The co-culture produced a wine with an intermediate glycerol content of 2.93 g/l. At the end of fermentation, the dominance of *S*. *cerevisiae* strains (Figures 1, 2) made uniform all the wines with a glycerol content of about 10 g/l and an ethanol concentration of about of 14% (v/v). Similar behaviors were observed for other parameters such as volatile acidity, pH, TTA, and organic acids concentration. In all the samples the consumption of malic acid started before alcoholic fermentation was completed. This fact could be related to an high number of malolatic bacteria on grapes, as reported by Renouf et al. (2006), who found *Oenococcus oeni* and other lactic acid bacteria at the beginning of alcoholic fermentation. On the other hand, Nehme et al. (2010) reported simultaneous fermentations by inoculated yeasts and malolactic acid bacteria. Obtained wines were also analyzed for the presence of BA. In all wines cadaverine, tryptamine, β- phenylethylamine, tyramine, and histamine were below the limit of detection for the method used (Manetta et al., 2016). The levels of ethanolamine, ethylamine, isoamilamine, and putrescine had no significant changes. Their content was quite similar in all samples with ethanolamine which was the most abundant amine found ranging from 21 to 24 mg/l (data not shown).

**Table 1:** **Montepulciano d'Abruzzo wine characteristics fermented with autochthonous ***S. cerevisiae*** strains (SRS1 and RT73), commercial strain (CS), co-culture (SRS1+STS12) and non-***Saccharomyces*** strains (STS12 and STS45) after 3 and 15 days (in parentheses) of fermentation**.

**Parameters**	**Strains**					
	**CS**	**SRS1**	**RT73**	**SRS1+STS12**	**STS12**	**STS45**
Ethanol^*^ (% v/v)	0.36±0.18^a^	0.41±0.08^a^	0.91±0.68^a^	2.44±1.69^b^	2.77±0.15^bc^	2.05±0.09^c^
	(14.40±0.04)^d^	(14.26±0.19)^d^	(14.24±0.08)^d^	(14.21±0.08)^d^	(14.24±0.13)^d^	(14.10±0.11)^d^
Reducing sugar (g/l)	215±10^a^	220±14^a^	197±17^a^	166±34^ab^	167±12^b^	174±12^b^
	(2.07±0.13)^c^	(2.18±0.28)^c^	(2.06±0.22)^c^	(1.81±0.10)^d^	(1.96±0.14)^d^	(1.95±0.12)^d^
pH	3.29±0.09^a^	2.94±0.61^a^	3.33±0.05^a^	3.36±0.01^a^	3.33±0.10^a^	3.35±0.09^a^
	(3.30±0.02)^a^	(3.34±0.04)^a^	(3.31±0.04)^a^	(3.33±0.06)^a^	(3.33±0.07)^a^	(3.31±0.10)^a^
Volatile acidity^**^ (g/l)	0.07±0.04^a^	0.05±0.02^a^	0.09±0.02^ab^	0.14±0.06^bc^	0.14±0.02^c^	0.16±0.05^c^
	(0.47±0.06)^d^	(0.58±0.15)^d^	(0.55±0.12)^d^	(0.51±0.08)^d^	(0.57±0.09)^d^	(0.49±0.09)^d^
Titratable acidity^***^ (g/l)	6.03±0.30^a^	5.97±0.33^a^	5.89±0.14^a^	6.10±0.19^a^	6.20±0.21^a^	6.27±0.22^a^
	(7.02±0.19)^b^	(6.55±0.26)^b^	(6.80±0.18)^b^	(6.78±0.38)^b^	(7.02±0.27)^b^	(7.48±0.13)^c^
Citric acid (g/l)	0.15±0.01^a^	0.06±0.01^b^	0.14±0.02^a^	0.20±0.03^c^	0.19±0.04^bc^	0.27±0.04^c^
	(0.59±0.09)^de^	(0.55±0.02)^d^	(0.57±0.07)^d^	(0.59±0.03)^d^	(0.63±0.04)^e^	(0.62±0.05)^de^
Lactic acid	0.15±0.08^a^	0.30±0.05^b^	0.15±0.02^a^	0.03±0.01^c^	−	0.16±0.02^a^
	(1.10±0.22)^d^	(1.04±0.11)^d^	(1.15±0.19)^d^	(1.14±0.13)^d^	(1.01±0.17)^d^	(0.77±0.15)^e^
Malic acid (g/l)	1.00±0.10^a^	1.08±0.21^ab^	1.17±0.06^b^	1.18±0.09^b^	1.20±0.08^b^	1.17±0.12^ab^
	(0.34±0.21)^cde^	(0.10±0.04)^c^	(0.21±0.09)^cd^	(0.21±0.08)^cd^	(0.39±0.11)^d^	(0.55±0.12)^de^
Tartaric acid (g/l)	6.88±0.21^ab^	7.22±0.38^a^	6.78±0.31^ab^	6.59±0.38^b^	6.79±0.21^ab^	6.30±0.19^b^
	(3.0±0.06)^c^	(2.98±0.18)^c^	(3.11±0.27)^c^	(2.87±0.27)^c^	(2.96±0.17)^c^	(2.99±0.15)^c^
Glycerol (g/l)	2.2±1.5^ab^	1.38±0.19^a^	1.67±0.65^a^	2.9±1.2^b^	3.39±0.27^b^	3.02±0.24^b^
	(10.96±0.28)^c^	(10.12±0.08)^d^	(10.0±0.17)^d^	(10.16±0.41)^d^	(10.22±0.19)^d^	(10.71±0.54)^d^

* *ml of alcohol/100 ml of wine*,

** *expressed as acetic acid*,

*** *expressed as tartaric acid*.

*Data are expressed as average ± SD. Same letters indicate samples in the same line with non-significant differences (p < 0.05)*.

### Volatile compounds

**Figure 3:**
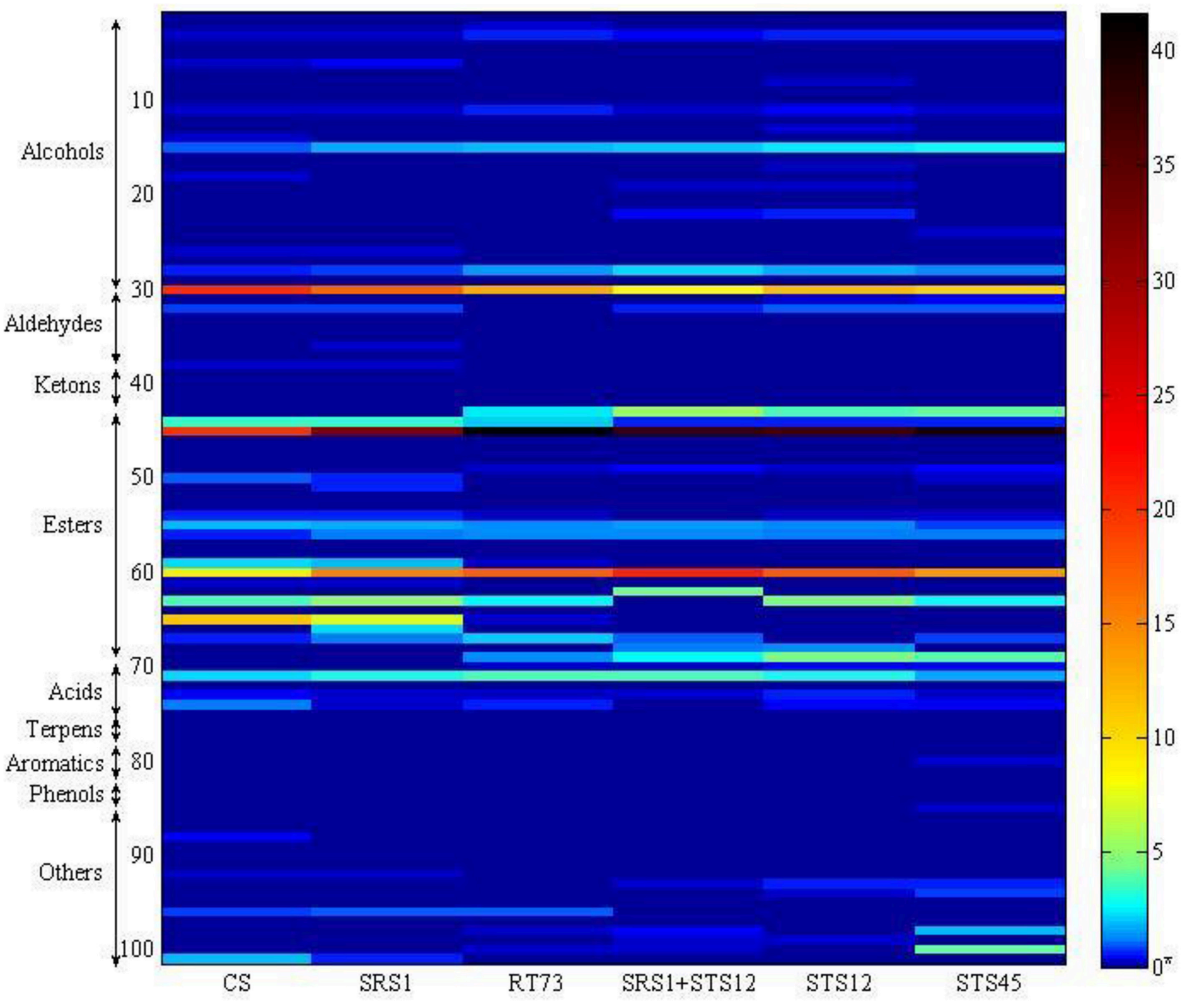
**Heatmap representing volatile profile of autochthonous ***S. cerevisiae*** strains (SRS1 and RT73), commercial strain (CS), co-culture (SRS1+STS12), and non-***Saccharomyces*** strains (STS12 and STS45)**. Compounds were organized by chemical families, and with the indication of the number of compounds per family. Each line corresponds to one metabolite, and each column corresponds to each strain. For the correspondence between number and volatile compound see Table 2. ^*^The quantitative analysis of wine aroma compounds was carried out on the basis of the relative peak area (Qi) calculated from head space SPME (HS/SPME) gas chromatograms after addition of known amounts of analyte standards.

The volatile metabolites of the Montepulciano d'Abruzzo wines obtained with autochthonous strains of *S*. *cerevisiae* and NS and a mixed culture have been identified for a total of 101. Aroma compounds belonged to eight different families such as alcohols, aldehydes, ketones, esters, acids, terpenes, phenols, and aromatic compounds. The number of metabolites ranged from 63 for the wine produced by strain SRS1, 53 by STS45, 51 by CS, 49 by RT73, and mixed culture SRS1+STS12 and 47 by STS12 (Table 2). Table 2 shows the main volatile molecules identified in relation to starter culture used. In the table only the main components of each aroma profile are reported. Nevertheless their presence represented at least the 95% of the total area in all the wine. Esters represented the major group for all the wines, followed by alcohols. The wine obtained with the co-culture showed the lowest relative percentage of alcohols in the heat space (about 14%), while those produced with *S*. *cerevisiae* CS and SRS1 were characterized by the highest ones, about 24.78 and 28.08%, respectively. In particular 2-phenylethanol (line 30, rose odor) had the highest relative percentage, ranging from 8.07% (SRS1+STS12) to 21.2% (CS). Differences were observed also for 2-methyl-1-propanol (line 15) and 1-hexanol (line 28, fruity and erbal odor) prevailing in wines fermented by NS and co-culture. Regarding esters the relative percentage in the heat space ranged from 57.42% (CS) to 77.38% (mixed culture), with more differences on the relative quantities of compounds among strains, as it can be easily evaluated from Figure 3 built in order to better visualize the wine characterizing volatile molecules in relation to the starter used. The main esters present in the wines were 3-methylbut-1-yl methanoate (line 45, fruit aroma), ranging from 19.97% (CS) to 41.7% (RT73) followed by ethyl ethanoate (line 60), due to the large quantities of ethanol present. Isoamyl acetate was produced in relevant quantities only by the *S*. *cerevisiae* strains (line 44, banana aroma), whereas ethyl octanoate (line 65) and ethyl decanoate (line 59), generally associated to fruity aroma, were produced only by CS and SRS1. Ethyl hexanoate (line 63), related to red apple, fruity apple or estery flavor was completely absent in the co-culture wine. Acids ranged from 2.8% (STS45) to 4.73% (RT73). The other compounds were present only in low amount or absent (Table 2).

**Table 2:** **Main volatile compounds identified (expressed as percentage of the peak area of each compound compared to the total area) ad in the wines produced by ***S. cerevisiae*** and non-***Saccharomyces*** strains**.

**Line**	**Compounds**	**CS**	**SRS1**	**RT73**	**SRS1+STS12**	**STS12**	**STS45**
**ALCOHOL**							
1^*^	(2E)-3,7-dimethylocta-2,6-dien-1-ol	–	0.1 ± 0.09	–	–	–	–
2	3,7,11-Trimethyl-1,6,10-dodecatrien-3-ol	–	0.07 ± 0.01	0.2 ± 0.09	–	–	–
3	1-butanol	0.3 ± 0.0	0.3 ± 0.1	0.7 ± 0.11	0.53 ± 0.05	0.6 ± 0.1	0.6 ± 0.02
4	1-dodecanol	–	0.07 ± 0.05	–	–	–	–
5	1-nonanol	–	–	0.03 ± 0.02	–	–	–
6	1-octanol	0.37 ± 0.15	0.4 ± 0.1	–	–	–	–
7	Oct-1-en-3-ol	–	0.03 ± 0.01	0.07 ± 0.03	0.1 ± 0.01	0.1 ± 0.02	0.1 ± 0.01
8	1-pentanol	–	–	0.07 ± 0.01	0.17 ± 0.02	0.2 ± 0.01	0.1 ± 0.01
9	1-undecanol	0.03 ± 0.01	–	–	–	–	–
10	2,2 ethoxyethoxy ethanol	–	–	–	–	0.1 ± 0.01	–
11	2,3-butandiol	0.35 ± 0.10	0.27 ± 0.11	0.7 ± 0.11	0.3 ± 0.1	0.5 ± 0.02	0.2 ± 0.02
12	2,3-dimethyl-2-hexanol	–	0.1 ± 0.03	–	–	–	–
13	2-decen-1-ol	–	–	–	–	0.2 ± 0.02	0.1 ± 0.01
14	2-ethyl-1-hexanol	0.2 ± 0.05	0.17 ± 0.03	–	–	–	–
15	2-methyl 1-propanol	0.97 ± 0.12	1.73 ± 0.60	1.93 ± 0.77	2.17 ± 0.15	2.5 ± 0.7	2.8 ± 0.8
16	2-octanol	–	–	–	0.13 ± 0.05	0.1 ± 0.01	0.1| ± 0.01
17	2-pentanol	–	–	0.03 ± 0.01	0.13 ± 0.01	0.2 ± 0.01	–
18	3,4-dimethyl-2-hexanol	0.23 ± 0.09	0.1 ± 0.04	0.03 ± 0.01	0.07 ± 0.02	–	–
19	3-hexen-1-ol	–	0.07 ± 0.01	0.13 ± 0.04	0.2 ± 0.08	0.2 ± 0.01	0.1 ± 0.01
20	3-methyl-1-pentanol	–	–	0.03 ± 0.01	0.07 ± 0.01	0.1 ± 0.01	–
21	3-(methylthio)-1-propanol	0.07 ± 0.01	0.03 ± 0.01	0.03 ± 0.01	–	–	–
22	5-methyl-2-hexanol	–	–	0.03 ± 0.01	0.4 ± 0.12	0.7 ± 0.03	0.1 ± 0.01
23	5-methoxy-1-pentanol	0.17 ± 0.07	0.07 ± 0.02	–	–	–	–
24	6,10,13-trimethyl-1-tetradecanol	0.03 ± 0.01	0.03 ± 0.01	–	–	–	0.2 ± 0.01
25	3,7-dimethyloct-6-en-1-ol	–	0.1 ± 0.02	–	–	–	–
26	Phenylmethanol	0.23 ± 0.06	0.23 ± 0.04	0.13 ± 0.04	0.13 ± 0.05	0.1 ± 0.01	0.1 ± 0.01
27	2-ethoxyethanol	–	–	0.03 ± 0.01	–	–	–
28	1-hexanol	0.63 ± 0.05	0.83 ± 0.32	1.5 ± 0.7	2.27 ± 0.15	1.7 ± 0.7	1.3 ± 0.6
29	1-heptanol	–	0.07 ± 0.01	–	–	–	–
30	2-phenylethanol	21.2 ± 7.59	16.03 ± 5.72	12.77 ± 5.98	8.07 ± 1.59	11.7 ± 2.5	10.4 ± 2.68
Total		24.78	20.80	18.41	14.74	19.0	16.2
**ALDEHYDES**							
31	3-furaldehyde	–	–	–	0.1 ± 0.01	0.3 ± 0.08	0.4 ± 0.2
32	Benzaldehyde	0.83 ± 0.05	0.87 ± 0.15	0.17 ± 0.05	0.63 ± 0.15	1.0 ± 0.3	1.0 ± 0.33
33	2-Phenylacetaldehyde	–	–	–	–	–	0.1 ± 0.03
34	Carbaldeide	–	–	–	–	0.1 ± 0.01	0.1 ± 0.02
35	Decanal	0.17 ± 0.02	0.17 ± 0.08	–	–	–	–
36	Furan-2-carbaldehyde	0.17 ± 0.05	0.23 ± 0.05	0.07 ± 0.01	0.07 ± 0.01	–	–
37	Heptanal	0.07 ± 0.01	–	–	–	–	–
38	Nonanal	0.27 ± 0.01	0.3 ± 0.16	–	–	–	–
Total		1.51	1.57	0.24	0.80	1.4	1.6
**KETONS**							
39	2,3-butanedione	–	–	0.17 ± 0.0.01	0.13 ± 0.01	–	–
40	(E)-1-(2,6,6-Trimethyl-1-cyclohexa-1,3-dienyl)but-2-en-1-one	0.1 ± 0.01	0.07 ± 0.01	–	–	–	0.1 ± 0.01
41	3-hexanone	–	–	0.03 ± 0.01	0.07 ± 0.01	0.1 ± 0.01	0.1 ± 0.03
42	3-hydroxy-2-butanone	–	0.07 ± 0.01	0.03 ± 0.01	0.07 ± 0.01	0.1 ± 0.01	0.1 ± 0.02
**Total**		0.1	0.14	0.23	0.27	0.2	0.3
**ESTERS**							
43	2-methylbut-1-yl ethanoate	–	–	2.53 ± 0.38	5.4 ± 0.52	3.8 ± 0.7	4.1 ± 1.59
44	3-methylbut-1-yl ethanoate	3.37 ± 0.8	3.47 ± 1.02	2.13 ± 0.73	0.63 ± 0.23	0.6 ± 0.2	0.6 ± 0.23
45	3-methylbut-1-yl methanoate	19.97 ± 1.05	31.1 ± 11.19	41.7 ± 2.68	37.33 ± 3.13	36.4 ± 3.78	39.8 ± 3.89
46	Ethyl furan-2-carboxylate	0.07 ± 0.01	0.13 ± 0.01	–	–	0.1 ± 0.01	–
47	Pentan-2-yl methanoate	–	0.03 ± 0.01	–	–	–	–
48	3,7-dimethyloct-6-enyl methanoate	0.07 ± 0.01	–	–	–	–	–
49	2-methylpropyl acetate	–	–	0.23 ± 0.1	0.43 ± 0.05	0.2 ± 0.04	0.4 ± 0.2
50	2-phenylethyl ethanoate	1.0 ± 0.2	0.73 ± 0.16	0.13 ± 0.05	0.1 ± 0.01	0.1 ± 0.01	0.2 ± 0.1
51	Hexyl ethanoate	0.07 ± 0.01	0.67 ± 0.26	–	–	–	–
52	Ethyl phenylacetate	0.1 ± 0.01	0.1 ± 0.01	–	–	–	–
53	Ethyl 3-phenylpropanoate	–	0.07 ± 0.01	–	–	–	–
54	Benzyl 2-hydroxybenzoate	0.6 ± 0.1	0.67 ± 0.17	0.27 ± 0.15	0.17 ± 0.05	0.2 ± 0.03	0.2 ± 0.08
55	Butanedioic acid, diethyl ester	2.0 ± 0.26	1.8 ± 0.40	1.3 ± 0.78	1.5 ± 0.65	1.3 ± 0.5	0.9 ± 0.03
56	Ethyl butanoate	0.63 ± 0.05	1.23 ± 0.41	1.37 ± 0.11	1.3 ± 0.12	1.2 ± 0.37	1.2 ± 0.2
57	Ethyl 2-methylbutanoate	0.1 ± 0.01	0.1 ± 0.01	0.07 ± 0.01	0.1 ± 0.01	0.1 ± 0.02	0.1 ± 0.04
58	Ethyl 3-methylbutanoate	0.07 ± 0.01	0.1 ± 0.01	0.07 ± 0.01	0.1 ± 0.01	0.1 ± 0.01	0.1 ± 0.02
59	Ethyl decanoate	3.37 ± 0.51	1.03 ± 0.56	0.33 ± 0.2	0.13 ± 0.05	–	–
60	Ethyl ethanoate	9.07 ± 0.92	15.07 ± 2.44	17.13 ± 1.44	20.93 ± 4.66	17.6 ± 3.54	13.9 ± 2.73
61	Ethyl heptanoate	0.2 ± 0.01	0.3 ± 0.08	–	–	–	0.1 ± 0.02
62	3-Oxohexanedioic Acid Diethyl Ester	–	0.03 ± 0.01	–	4.3 ± 1.08	–	–
63	Ethyl hexanoate	3.73 ± 0.75	5.03 ± 2.49	2.73 ± 1.08	–	4.6 ± 1.79	2.8 ± 0.74
64	methyl 3-metoxy-aminopropanoate	–	–	0.03 ± 0.01	–	–	–
65	Ethyl octanoate	12.27 ± 0.97	7.0 ± 1.94	0.3 ± 0.1	–	–	–
66	Methyl octanoate	–	2.37 ± 0.98	–	–	–	–
67	Ethyl 2-hydroxypropanoate	0.73 ± 0.31	1.2 ± 0.85	2.13 ± 0.97	1.1 ± 0.9	–	0.8 ± 0.06
68	Ethyl 2-methylpropanoate	–	0.07 ± 0.01	–	1.23 ± 0.78	1.3 ± 0.75	–
69	Ethyl undecanoate	–	–	1.3 ± 0.86	2.63 ± 1.1	4.6 ± 1.23	4.0 ± 1.35
Total		57.42	71.30	73.75	77.38	72.20	69.2
**ACIDS**							
70	3-methyl butanoic acid	–	–	0.23 ± 0.09	0.3 ± 0.02	0.4 ± 0.03	0.4 ± 0.11
71	Acetic acid	2.27 ± 0.47	3.03 ± 1.05	3.73 ± 1.71	3.77 ± 1.19	3.0 ± 1.25	1.7 ± 0.64
72	4-hydroxy-butanoic acid	–	0.07 ± 0.01	–	–	–	–
73	Hexanoic acid	0.53 ± 0.2	0.23 ± 0.05	0.2 ± 0.03	0.23 ± 0.05	0.6 ± 0.05	0.3 ± 0.17
74	Octanoic acid	1.2 ± 0.75	0.33 ± 0.1	0.57 ± 0.13	0.1 ± 0.01	0.5 ± 0.06	0.4 ± 0.09
75	Propanoic acid	0.07 ± 0.01	–	–	–	–	–
Total		4.07	3.66	4.73	4.40	4.50	2.8
**TERPENS**							
76	3,7,7-trimethylbicyclo[4.1.0]hept-3-ene	0.07 ± 0.02	–	–	–	–	–
77	2-(4-Methyl-1-cyclohex-3-enyl)propan-2-ol	–	0.03 ± 0.01	–	–	–	–
Total		0.07	0.03				
**AROMATICS**							
78	Phenylethene	0.07 ± 0.03	0.13 ± 0.08	–	0.03 ± 0.02	0.1 ± 0.03	0.1 ± 0.02
79	1-methyl-4-(1-methylethyl)benzene	0.17 ± 0.08	0.1 ± 0.01	–	–	–	0.1 ± 0.01
80	1,3 dymethyl, 2-ethyl benzene	–	–	–	–	0.1 ± 0.02	0.3 ± 0.04
81	Toluene	–	–	–	–	–	0.1 ± 0.02
82	Dithiolane	–	–	–	–	0.1 ± 0.05	0.1 ± 0.04
Total		0.24	0.23		0.03	0.3	0.7
**PHENOLS**							
83	4,4′-(propane-2,2-diyl)diphenol	–	0.1 ± 0.01	–	–	–	–
84	4-methyl phenol	–	0.1 ± 0.02	–	–	–	0.1 ± 0.01
Total			0.2				0.1
**OTHERS**							
85	1-methoxy octane	–	–	–	0.07 ± 0.01	–	0.2 ± 0.01
86	1-chlorooctane	–	0.07 ± 0.01	0.17 ± 0.08	–	–	–
87	1-methoxy-2-methyl-propane	0.13 ± 0.05	–	–	0.17 ± 0.02	–	–
88	4-amino-1,2,4-triazole	0.4 ± 0.1	–	–	–	–	–
89	Pentamine	0.13 ± 0.01	0.03 ± 0.02	–	–	–	–
90	2-pentylfuran	0.03 ± 0.01	0.07 ± 0.03	–	–	–	–
91	3-heptene	–	–	–	–	–	0.1 ± 0.01
92	Ciclo-heptane	0.27 ± 0.05	0.2 ± 0.17	–	0.13 ± 0.01	–	–
93	Decamethyl cyclopentasiloxane	–	–	–	0.23 ± 0.1	0.6 ± 0.09	0.6 ± 0.13
94	2-heptamethyl nonene	–	–	–	0.17 ± 0.08	0.3 ± 0.1	0.9 ± 0.09
95	5H-dibenzo[b,f]azepine	–	–	–	–	–	0.1 ± 0.02
96	Silane	1.4 ± 0.31	0.1 ± 0.43	1.0 ± 0.73	–	–	–
97	Indole	–	–	0.03 ± 0.02	–	–	–
98	Thiolane	–	–	0.2 ± 0.01	0.4 ± 0.07	0.1 ± 0.07	1.9 ± 0.8
99	Dihydrofuran-2(3H)-one	–	–	0.17 ± 0.07	0.3 ± 0.01	0.3 ± 0.02	0.1 ± 0.09
100	2-thiophene acetic acid	–	–	0.27 ± 0.09	0.37 ± 0.12	0.1 ± 0.04	4.2 ± 1.33
101	3-thiopheneethanol	2.4 ± 0.6	0.63 ± 0.25	–	–	–	–
Total		4.76	1.1	1.84	1.84	1.4	8.1

* *, metabolite number corresponding in the heatmap*.

**Figure 4:**
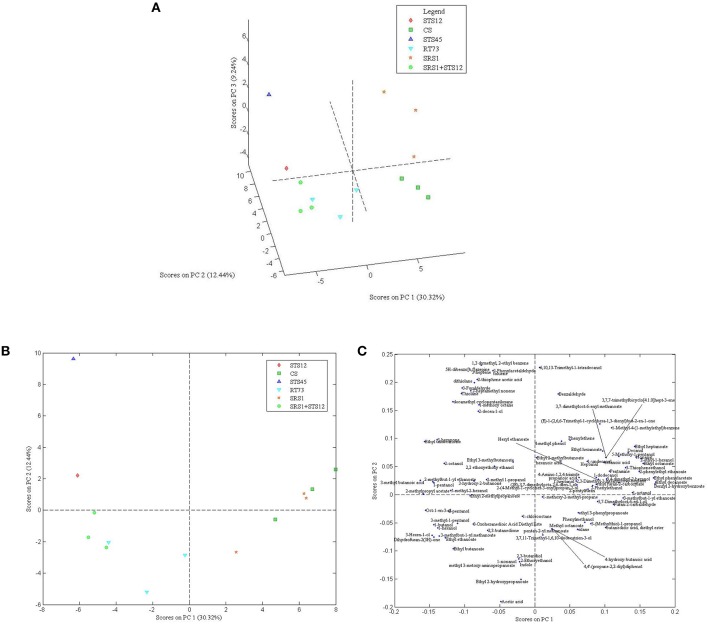
**Score plot of the first 3 PCs (A), score (B) and loading plot (C) of the first and second PCs after PC analysis on volatile compounds GC/MS-SPME data for autochthonous ***S. cerevisiae*** strains (SRS1, RT73), commercial strain (CS), non-***Saccharomyces*** strains (STS12 and STS45), and co-culture (SRS1+STS12)**.

In order to understand the variability among the strains, 101 aroma compounds data were submitted to PCA analysis (Figures 4A–C) to generate a visual representation of the wine discrimination on the basis of the specific aroma profiles generated by the strains used. The first three principal components were able to explain >50% of the total variances. Wines showed similar aroma profiles with differences for some compounds as reported above. The first 3 PCs score plot (Figure 4A) highlighted an overlapping of wines produced with SRS1+STS12, STS12, and RT73, while wines obtained with CS, SRS1, and STS45 were well differentiated. For a clearer comprehension of the loadings plot (Figure 4C), only the first 2 PCs of it were reported along with the first 2 PCs scores plot (Figure 4B). When collapsing the scores plot in two dimensions, separations between the different strains remained the same, except for an overlapping of CS and SRS1. Looking at the loading plot it was impossible to select a single class of compounds as the most important for a specific yeast (even observing the 3^rd^ component, data not shown). However, *H*. *uvarum* STS45 was characterized by sulfur compounds (thiolane and 2-thiophene-acetic acid), some aromatic compounds (2-phenylacetaldeide, 5H-dibenzo[b,f]azepine, toluene and 1,3 dimethyl, 2-ethyl benzene) and hydocarbons such as 3-heptene and 2-heptamethyl nonene. Moreover, most of aldehydes such as heptanal, nonanal, and decanal can be found in the first quadrant of the loading plot correlated with CS and SRS1. Most of alcohols with even number of C atoms such as 1-butanol, 1-hexanol, and 3-mehyl 1 pentanol were present in the 3^rd^ quadrant related to RT73 and to the co-culture SRS1+STS12.

### Sensory analysis

**Figure 5:**
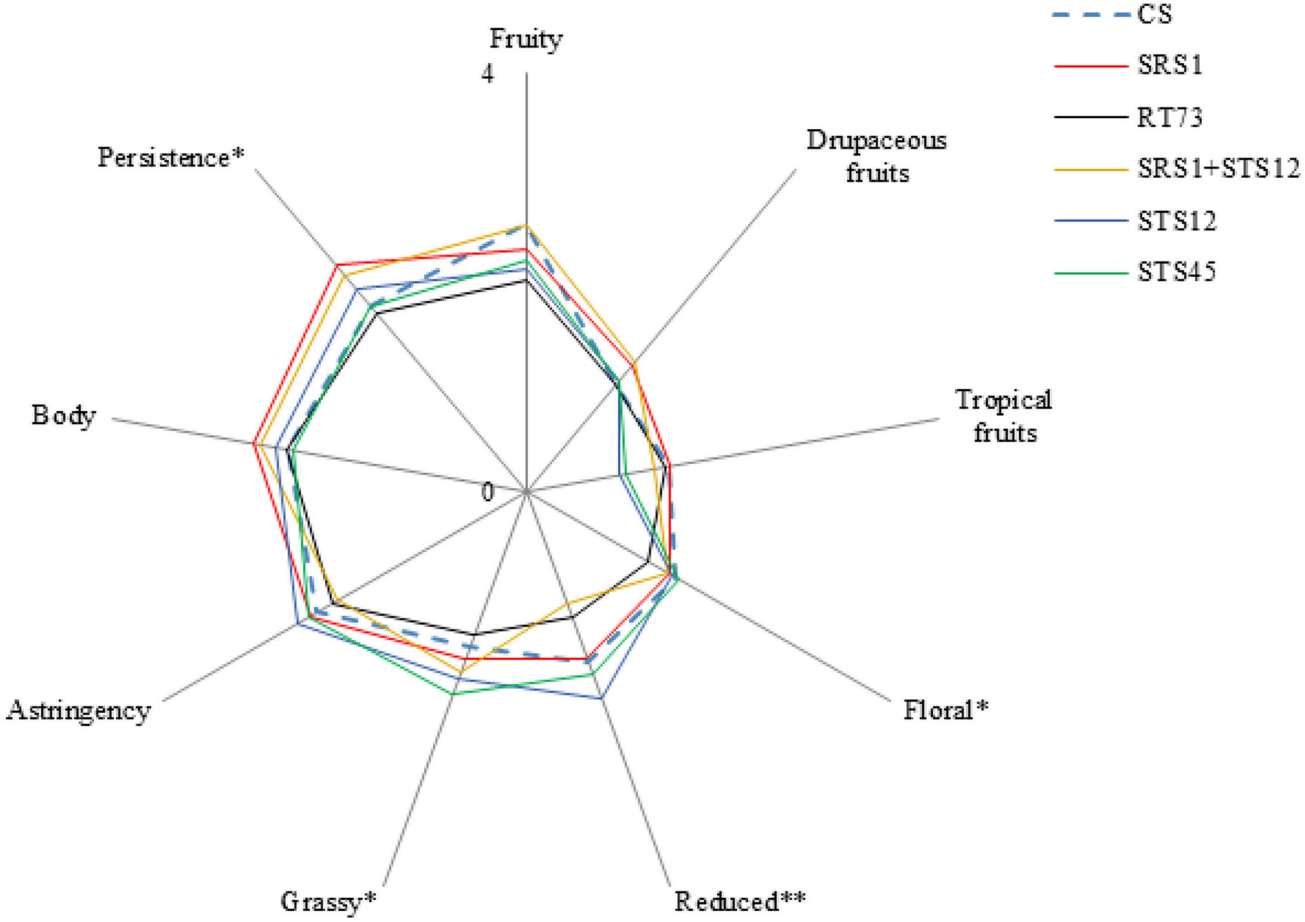
**Descriptive analysis of obtained wines**. ^*^p < 0.05, ^**^p < 0.01.

Sensory analysis revealed the influence of yeast strains on some of the considered descriptors. The wines fermented with SRS1 and the co-culture were characterized by a good floral and a highest persistence (Figure 5). Moreover negative attributes such as reduced and grassy were not very pronounced (significantly lower compared to STS12 and STS45 respectively). In particular, the co-culture had the lowest reduced aroma of all theses. Wines obtained with STS12 and STS45 were mainly characterized by grassy and reduced aroma. RT73 produced balanced wines with negative and positive attributes arranged in good proportions. Wines fermented with CS presented significantly low persistence, unwanted characteristic for Montepulciano d'Abruzzo wine. However these wines showed good aroma descriptors. In general, sensory analysis highlighted that the most interesting wines were those produced with SRS1 and the co-culture since they were characterized by a good floral, a highest persistence and, above all, have the reduced and grassy not too marked, as often it happens also in high quality Montepulciano d'Abruzzo wines.

## Discussion

Montepulciano d'Abruzzo is a native grape variety of *V. vinifera* L., grown in central Italy and used for production of high quality red wines. Limited studies have been carried out to improve its enological characteristics through the use of indigenous wine yeasts. The interest for autochthonous strains as single or mixed cultures in combination with *S. cerevisiae* is gaining more and more importance since they are potentially associated to a particular terroir and therefore adapted to a specific grape must reflecting the biodiversity of a particular area (Bokulich et al., 2014; Capozzi et al., 2015). For this reason, the application of indigenous mixed non-*Saccharomyces*/*Saccharomyces* starter, able to mimic wine biodiversity, could be a valid alternative to spontaneous fermentations, since the multi-starter ability to increase the organoleptic properties of wine and to minimize the microbial spoilage (Comitini et al., 2011; Ciani and Comitini, 2015).

In this study the organoleptic properties of Montepulciano d'Abruzzo wine and the fermentation of two indigenous strains of *S. cerevisiae* (SRS1, RT73), a strain of *S. bacillaris* (STS12), one of *H. uvarum* (STS45), and a co-culture of *S. cerevisiae* (SRS1) and *S. bacillaris* (STS12) were evaluated. The data highlighted that at 3 days faster fermentations were obtained in the musts inoculated with NS yeasts, in agreement with other authors (Mendoza et al., 2007; Fleet, 2008; Ciani et al., 2010; Suzzi et al., 2012b). Also the co-culture SRS1+STS12 showed a good fermentation kinetic in comparison with SRS1. The positive interaction between *S. cerevisiae* and *S. bacillaris* has been highlighted by other authors (Rantsiou et al., 2012; Suzzi et al., 2012b). The sugar consumption was faster in SRS1+STS12 co-culture than in *S. cerevisiae* pure cultures probably because of the osmotolerant and fructophilic character of this non-*Saccharomyces* yeast. In fact, it consumes sugars at the early stage of the fermentation, alleviating the *S. cerevisiae* from the osmotic stress, thereby improving also the fermentation kinetics (Rantsiou et al., 2012; Englezos et al., 2015). *H. uvarum* STS45 showed a good fermentation kinetic at the beginning however at the end of fermentation it showed the lowest viable count values. The disappearance of *Hanseniaspora* yeasts can be associated to their low ethanol tolerance or to the production of other toxic compounds besides ethanol (Egli et al., 1998; Fleet, 2003). *S. bacillaris* STS12 showed better fermentation kinetic than STS45 and a higher number of viable cells at the end of fermentation. Some authors reported that *S. bacillaris* was able to complete Macabeo must fermentation even if with a slight delay compared to the *S*. *cerevisiae* fermentation (Andorrà et al., 2010).

The enological parameters during the first days of fermentation highlighted the metabolic cooperation between inoculated and indigenous strains, although at the end of fermentation all wines showed similar characteristics due to the dominance of *S. cerevisiae* strains. In fact, also wines inoculated with NS wine yeasts showed low values of residual sugar and an ethanol concentration of about 14%, probably due to the contribution of indigenous *Saccharomyces* population present in the must at the start of fermentation. The wine organoleptic properties are related to the presence of several compounds deriving from the yeast metabolism (Capozzi et al., 2015) and the dominance or competitiveness of a starter strain could have an influence on the sensorial quality of wine by imposing its aromatic profile or deleting the collaborative role of natural *S. cerevisiae* populations. In this study the microsatellites analysis performed directly on the must allowed to establish the dominance of all *S. cerevisiae* strains (SRS1, RT73, and CS) during all the fermentation process shaping wine aroma and the presence of other non-starter yeasts during fermentation with NS strains. In *S*. *cerevisiae*, microsatellites have been described as abundant and highly polymorphic in length (Richards et al., 2009), and for this reason, they are used as a reproducible and portable typing method (Hennequin et al., 2001; Schuller et al., 2004; Bradbury et al., 2005; Legras et al., 2005; Tofalo et al., 2013).

In all wines, the volatile acidity was below the legal limit of 1.2 g/l of acetic acid (Office Internationale de la Vigne et du Vin, 2009), since higher values can confer to wine a detrimental acidic flavor (Bely et al., 2003). In this context it is interesting to underline that despite acetic acid production is considered as a common pattern in apiculate yeasts (Romano et al., 2003), we found that wines inoculated with *H*. *uvarum* STS45 did not show an increased volatile acidity, in agreement with other authors (Andorrà et al., 2010; Suzzi et al., 2012b). In addition all wines showed low quantity of BA indicating the low decarboxylase activity of wine yeasts and indigenous malolactic bacteria (Marcobal et al., 2006; Smit et al., 2008; Suzzi et al., 2012b).

Esters were the most representative compounds in all wines according to Ferreira et al. (1995) and according to Suzzi et al. (2012a) the fruity character attributed to the aroma of Montepulciano wines is mainly related to apple, pear, and cherry notes. In fact, esters are a group of volatile compounds, arise from yeast metabolic activity, that impart a mostly pleasant smell (Capozzi et al., 2015). The wines produced with SRS1 and CS were well differentiated by other wines as shown by PCA and sensory analyses acquiring the aromatic fingerprinting of the strain.

Specific features were also shown by wines produced with STS45. These wines were characterized by the presence of sulfur compounds. Sulfur compounds have different sensory properties and, although most of them could negatively affect the wine aroma, they can also give a positive contribute by adding fruity notes (Swiegers and Pretorius, 2005).

The wines produced with RT73 and SRS1+STS12 clustered together in the PCA analysis, however sensory analysis revealed that wines obtained with the co-culture showed interesting olfactory and tasting properties such as fruity, good body, and persistence which are important characteristics for red wines. In addition the simultaneously malolactic and alcoholic fermentation suggested a possible impact of lactic acid bacteria on the final wines. In fact it is well known as the role of malolactic fermentation is more than a deacidification, affecting the quality of wine positively, such as volatile acids and negatively such BA production (Liu, 2002; Renouf et al., 2006). In all the wines the content of BA was lower than the detection limits, confirming that lactic acid bacteria vary on the production of these compounds (Lonvaud-Funel, 2001).

The data obtained in this study highlighted that the use of NS autochthonous yeasts positively influence wine aroma profile. In particular STS45 produced wines with a specific aroma fingerprinting. In conclusion the natural cultures applied in cellar vinification in this study can be considered as a useful tool that take the advantages of the spontaneous fermentation, enhancing the chemical and organoleptic characteristics of the wine and avoiding the risk of stuck fermentations and microbial contamination.

## Author contributions

Conceived and designed the experiments: GS, RT. Performed the experiments: GP, MS, PG, FP. Analyzed the data: MS, DP, GA, RL. Wrote the paper: GS, RT, MS.

### Conflict of interest statement

The authors declare that the research was conducted in the absence of any commercial or financial relationships that could be construed as a potential conflict of interest.
